# Nuclear expression and/or reduced membranousexpression of β-catenin correlate with poor prognosis in colorectal carcinoma: A meta-analysis: Erratum

**DOI:** 10.1097/MD.0000000000006779

**Published:** 2017-04-21

In the article, “Nuclear expression and/or reduced membranous expression of β-catenin correlate with poor prognosis in colorectal carcinoma: A meta-analysis”,^[1]^ which appeared in Volume 95, Issue 49 of *Medicine*, affiliation a was published incorrectly and should have appeared as Department of Surgical Oncology and Caner institute (Key Laboratory of Cancer Prevention & Intervention, National Ministry of Education, Provincial Key Laboratory of Molecular Biology in Medical Sciences), Second Affiliated Hospital, Zhejiang University School of Medicine, Hangzhou, China.
